# Relationship between surgical volume and outcomes in elective and acute cholecystectomy: nationwide, observational study

**DOI:** 10.1093/bjs/znac415

**Published:** 2022-11-24

**Authors:** My Blohm, Gabriel Sandblom, Lars Enochsson, Mats Hedberg, Mikael Franko Andersson, Johanna Österberg

**Affiliations:** Department of Clinical Sciences, Intervention and Technology, Karolinska Institutet, Stockholm, Sweden; Department of Surgery, Mora Hospital, Mora, Sweden; Center for Clinical Research, Uppsala University, Falun, Sweden; Department of Clinical Science and Education, South General Hospital, Karolinska Institutet, Stockholm, Sweden; Department of Clinical Sciences, Intervention and Technology, Karolinska Institutet, Stockholm, Sweden; Department of Surgical and Perioperative Sciences, Surgery, Umeå University, Umeå, Sweden; Department of Surgery, Sunderby Hospital, Luleå, Sweden; Department of Surgery, Mora Hospital, Mora, Sweden; Department of Clinical Science and Education, South General Hospital, Karolinska Institutet, Stockholm, Sweden; Department of Clinical Sciences, Intervention and Technology, Karolinska Institutet, Stockholm, Sweden; Department of Surgery, Mora Hospital, Mora, Sweden; Center for Clinical Research, Uppsala University, Falun, Sweden

## Abstract

**Background:**

High surgical volumes are attributed to improved quality of care, especially for extensive procedures. However, it remains unknown whether high-volume surgeons and hospitals have better results in gallstone surgery. The aim of this study was to investigate whether operative volume affects outcomes in cholecystectomies.

**Methods:**

A registry-based cohort study was performed, based on the Swedish Registry of Gallstone Surgery. Cholecystectomies from 2006 to 2019 were included. Annual volumes for the surgeon and hospital were retrieved. All procedures were categorized into volume-based quartiles, with the highest group as reference. Low volume was defined as fewer than 20 operations per surgeon per year and fewer than 211 cholecystectomies per hospital per year. Differences in outcomes were analysed separately for elective and acute procedures.

**Results:**

The analysis included 154 934 cholecystectomies. Of these, 101 221 (65.3 per cent) were elective and 53 713 (34.7 per cent) were acute procedures. Surgeons with low volumes had longer operating times (*P* < 0.001) and higher conversion rates in elective (OR 1.35; *P* = 0.023) and acute (OR 2.41; *P* < 0.001) operations. Low-volume surgeons also caused more bile duct injuries (OR 1.41; *P* = 0.033) and surgical complications (OR 1.15; *P* = 0.033) in elective surgery, but the results were not statistically significant for acute procedures. Low-volume hospitals had more bile duct injuries in both elective (OR 1.75; *P* = 0.002) and acute (OR 1.96; *P* = 0.003) operations, and a higher mortality rate after acute surgery (OR 2.53; *P* = 0.007).

**Conclusion:**

This study has demonstrated that operative volumes influence outcomes in cholecystectomy. The results indicate that gallstone surgery should be performed by procedure-dedicated surgeons at hospitals with high volumes of this type of benign surgery.

## Teaser

This registry-based cohort study demonstrated that operative volumes, both for the surgeon and the hospital, influence outcomes of cholecystectomies. The results indicated that gallstone surgery should be performed by procedure-dedicated surgeons at hospitals with high volumes of this type of benign surgery

## Introduction

Increased surgical volume is widely associated with improved quality of care, especially for more complicated procedures^1–7^. Accordingly, centralization of highly specialized procedures to tertiary referral centres has been discussed and implemented worldwide^6^. For more common and less complex procedures, such as cholecystectomies, the evidence for centralization is more ambiguous. Some previously published studies^8–10^ showed decreased readmission rates, shorter hospital stays, and decreased overall charges for high-volume surgeons, but argued against the benefit of centralization, because the rates of major complications, bile duct injuries, and mortality were the same, both in experienced and less experienced hands. However, other studies^11–13^ indicated that high-volume surgeons had better results and fewer complications, especially for patients with acute cholecystitis and other high-risk conditions.

The complication rate in both elective and acute cholecystectomies is considerable. In 2021, the overall complication rate in Sweden was 7.2 per cent for elective surgery and 10.6 per cent for acute surgery, and as high as 28.6 per cent for open and converted procedures^14^. If high-volume surgeons and high-volume hospitals have lower complication rates, centralization of gallbladder surgery to units with high volumes of cholecystectomies could be a way to further increase patient safety. However, the issue is complicated because laparoscopic cholecystectomy is one of the most common surgical procedures, and an important cornerstone of surgical education. Thus, a balance must be achieved between high volume and the need to further educate residents and younger surgeons in laparoscopic surgery.

The aim of this study was to investigate whether the surgeon’s and hospital’s annual operative cholecystectomy volume has an impact on surgical outcomes, with the aim of reducing the knowledge gap to optimal care for patients undergoing cholecystectomy.

## Methods

### Ethical considerations

This research was approved by the Regional Research Ethics Committee in Uppsala, Sweden (2017/022).

### Study design

The study was designed as a registry-based cohort study, using data from the Swedish Registry of Gallstone Surgery and Endoscopic Retrograde Cholangiopancreatography (GallRiks). The cohort was defined as all patients undergoing open and laparoscopic cholecystectomies registered between 1 January 2006 and 31 December 2019. The study report was structured in accordance with the STROBE reporting checklist^15^.

### Participants and variables

The cohort included all cholecystectomies performed for the indication of colic pain, polyps, and gallstone complications (cholecystitis, pancreatitis, cholangitis, common bile duct stones). Annual volumes for the lead operating surgeon and the registering hospital were calculated for each operation, based on the total volume in the preceding year. The procedures were categorized into volume-based quartiles, giving four subgroups in the outcome analysis. Differences between the groups were analysed in terms of duration of operation, surgical complications (bleeding, visceral perforation, bile duct injury, bile leakage, and abscess), bile duct injury, conversion to open surgery (or open surgery from the start), and 30-day postoperative mortality. Duration of operation was defined as the interval from the start of the procedure until wound closure. Elective and acute procedures (patients admitted acutely and operated on during the same hospital stay) were analysed separately. Patient age, sex, and ASA fitness grade were considered as potential confounders and adjusted for in the multivariable analysis.

### Data sources

GallRiks was founded in May 2005 by the Swedish National Board of Health, the Swedish Society of Laparoscopic Surgery, the Swedish Society of Upper Abdominal Surgery, and the Swedish Surgical Association. The registry is supported financially by the Swedish National Health Authorities. The main aim of the registry is to ensure uniform and evidence-based treatment of gallstone disease and high-quality patient care nationwide. In addition, the registry is a reliable source of continuously updated information about indications, outcomes, and the negative aspects of novel techniques. The registry has been described in detail previously^16^.

Approximately 12 000–14 000 cholecystectomies, in both children and adults, are registered every year and the national coverage of the registry is 94.5 per cent^14^. Each procedure is registered online by the surgeon, using their unique identification code, which remains constant, even if the surgeon is active at different hospitals. Thirty-day postoperative follow-up is undertaken by a local coordinator. The follow-up and registration of complications are based on information from the medical records. The registry includes patient characteristics, surgery-specific parameters, and information about intraoperative and postoperative adverse events. Intraoperative adverse events include bile duct injury (any lesion to the bile ducts other than the cystic duct), bleeding (requiring intervention, conversion to open surgery or blood transfusion), visceral perforation, and any other reason for the operation to be terminated prematurely. Postoperative adverse events are defined as any complication presenting during the first 30 days after operation. Thirty-day mortality data are retrieved from the National Population Registry.

### Bias

Registrations should be carried out online during or immediately after the operation, to reduce the risk of recall bias. The validity of the registry data is monitored by independent reviewers at least every third year. The validation process has shown a high degree of correctness (97 per cent) without missing serious adverse events^14,17^.

### Study size

All cholecystectomies performed in Sweden between 2006 and 2019 were included. The registry started in 2005, but did not reach adequate national coverage until 2006. Cholecystectomies as part of more extensive procedures, such as malignant surgery and transplant surgery, were not included in the data set. The goal was to include as many valid operations as possible to use the full potential of the national registry.

### Statistical analysis

Annual operative volumes for the lead surgeon and performing hospital were calculated from the number of cholecystectomies undertaken in the year preceding each procedure. The surgeon’s unique identification number was used when calculating individual volumes. Four volume groups, based on quartile individual and centre volumes, were used in the analysis. The highest quartile was used as reference category. Patient demographics were presented in contingency tables. The association between annual volume and duration of surgery was calculated using a mixed linear model with intercept of the surgeon nested in hospital as random effects, adjusting for age, sex, and ASA grade, with results presented as mean increase in duration of operation. The mixed linear model was selected for analysis of operating time to compensate for the vast time differences among the procedures. The associations between annual volumes and risk of surgical complications, bile duct injury, conversion to open surgery, and 30-day mortality were calculated using generalized estimated equations with an independent correlation structure and robust standard errors, adjusted for patient age, sex, and ASA grade; data are presented as ORs with 95 per cent confidence intervals. Only complete cases were analyzed in the models. *P* < 0.050 was considered significant. Spline functions with two knots at the 33rd and 67th percentiles were used to plot mean duration of operation, risk of surgical complications, bile duct injury, and conversion to open surgery with respect to operative volume, with pointwise 95 per cent confidence intervals. A logarithmic scale for operative volume was used in the diagrams.

Statistical analysis was performed with SPSS^®^ version 28.0 (IBM, Armonk, NY, USA) and diagrams were created with R version 4.1.0 (R Foundation for Statistical Computing, Vienna, Austria).

## Results

**Fig. 1:**
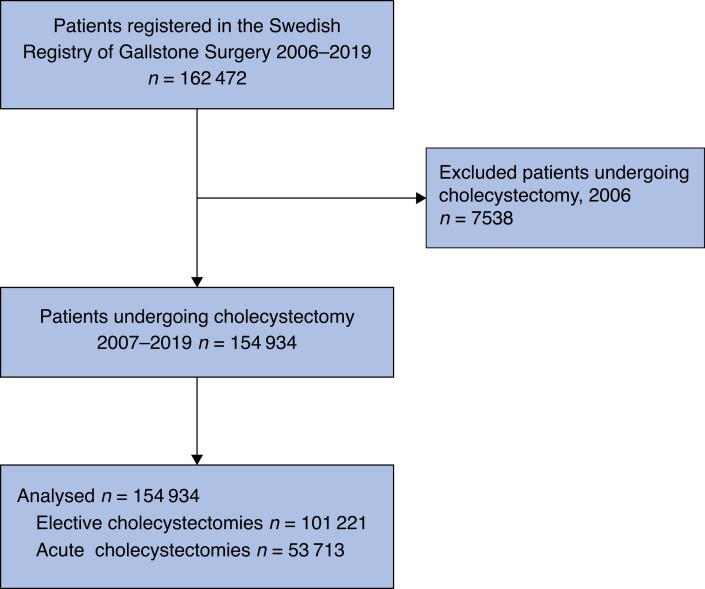
Study flow chart

During the 14 years from 1 January 2006 to 31 December 2019, a total of 162 472 patients were registered after undergoing laparoscopic or open cholecystectomy. The included operations were carried out by 2637 surgeons, from 89 different centres in Sweden. Cholecystectomies from 2006 were excluded from the analysis because no volumes could be calculated. This resulted in a database of 154 934 cholecystectomies, of which 101 221 (65.3 per cent) were elective and 53 713 (34.7 per cent) were acute procedures (*Fig. 1*).

Patient demographics are summarized in *Table 1*. The mean(s.d.) number of procedures undertaken during the preceding year was 24(23) (range 0–210) for the individual surgeon and 234(127) (range 0–515) for the hospital. Volume data were divided into quartiles for the analysis. For the individual surgeon, these were: quartile 1, 9 or fewer; quartile 2, 10–19; quartile 3, 20–33; and quartile 4, more than 33 cholecystectomies per year. Groups for the registering hospital were: quartile 1, 136 or fewer; quartile 2, 137–210; quartile 3, 211–305; and quartile 4, over 305 cholecystectomies per year. To facilitate presentation of the results, a cut-off between low and high volume was set between quartiles 2 and 3, giving a definition of low annual volume as fewer than 20 operations for the individual and fewer than 211 operations for the hospital. Of the 2637 surgeons, 1061 (40.2 per cent) carried out at least 20 procedures per year and 40 (44.9 per cent) of the 89 centres registered more than 210 operations per year. The follow-up time was 30 days, based on the organization of the registry.

**Table 1:** Demographics of included patients

	Elective surgery (*n* = 101 221)	Acute surgery (*n* = 53 713)
**Age (years)**		
ȃ< 50	31 273 (30.9)	16 220 (30.2)
ȃ≥ 50	69 854 (69.0)	37 250 (69.4)
ȃMissing	94 (0.1)	243 (0.4)
**Sex ratio (M : F)**	30 953 (30.6): 70 261 (69.4)	21 980 (40.9): 31 705 (59.0)
ȃMissing	7 (0.0)	28 (0.1)
**ASA fitness grade**		
ȃI	50 216 (49.6)	22 213 (41.4)
ȃII–III	43 579 (43.1)	24 793 (46.1)
ȃ≥ IV	7303 (7.2)	6302 (11.7)
ȃMissing	123 (0.1)	405 (0.8)
**Surgical approach**		
ȃLaparoscopic	93 568 (92.4)	42 340 (78.8)
ȃLaparoscopic converted to open	3864 (3.8)	5609 (10.5)
ȃOpen	2305 (2.3)	5324 (9.9)
ȃOther techniques	1484 (1.5)	440 (0.8)

Values are *n* (%).

### Cholecystectomy data

**Fig. 2:**
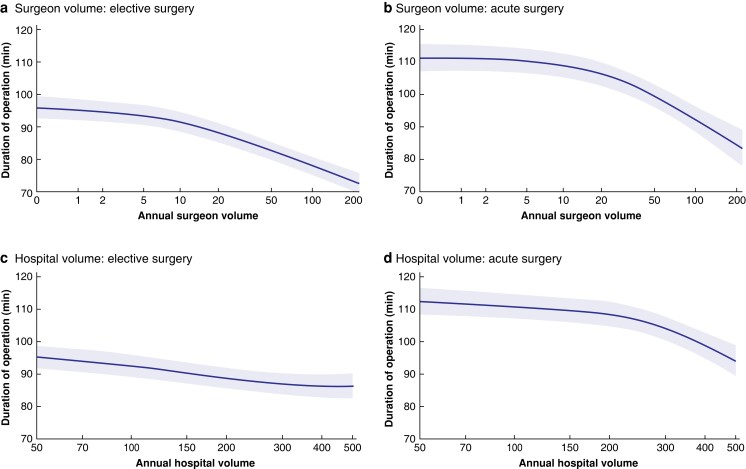
Spline functions for mean duration of operation for elective and acute procedures, with respect to operative volume for surgeons and hospitals Duration of operation for **a** elective and **b** acute operations according to surgeon volume, and **c** elective and **d** acute operations according to hospital volume. Shaded areas represent 95% confidence intervals.

The mean(s.d.) duration of surgery was 92(45) min for elective surgery and 115(55) min for acute surgery. The mean duration for the different volume categories is shown in *Table 2*. The operating time decreased significantly with increasing volumes for both the individual surgeon and the hospital, in both elective and acute surgery (*Table 2* and *Fig. 2*).

**Table 2:** Mixed-model analysis for surgeon and hospital volumes with duration of operation as outcome

Volume per year	Elective surgery‡			Acute surgery§		
	Duration of surgery (min)*	Increase†	*P*	Duration of surgery (min)*	Increase†	*P*
**Surgeon**						
ȃ≤ 9	103(44)	10.80 (9.85, 11.75)	<0.001	123(53)	10.29 (8.60, 11.98)	<0.001
ȃ10–19	97(45)	6.70 (6.10, 7.89)	<0.001	120(56)	8.04 (6.43, 9.66)	<0.001
ȃ20–33	89(44)	3.65 (2.85, 4.45)	<0.001	111(55)	3.59 (2.08, 5.09)	<0.001
ȃ> 33	80(42)	–		99(53)	–	
**Hospital**						
ȃ≤ 136	94(45)	4.41 (2.77, 6.06)	<0.001	120(54)	3.63 (0.92, 6.34)	0.009
ȃ137–210	95(46)	2.76 (1.26, 4.25)	<0.001	119(55)	1.99 (−0.47, 4.45)	0.113
ȃ211–305	93(45)	1.31 (−0.02, 2.62)	0.053	118(57)	4.65 (2.56, 6.75)	<0.001
ȃ> 305	85(42)	–		104(52)	–	

*Values are mean(s.d.); †values in parentheses are 95% confidence intervals. The analyses were adjusted for age, sex, and ASA grade. Some ‡235 and §662 patients were excluded owing to missing values.

Cholangiography was performed in 91.1 per cent of elective and 92.3 per cent of acute procedures, according to the standard routine in Sweden. Common bile duct stones were found in 7.5 per cent of elective and 19.1 per cent of acute cholecystectomies.

### Complications

**Fig. 3:**
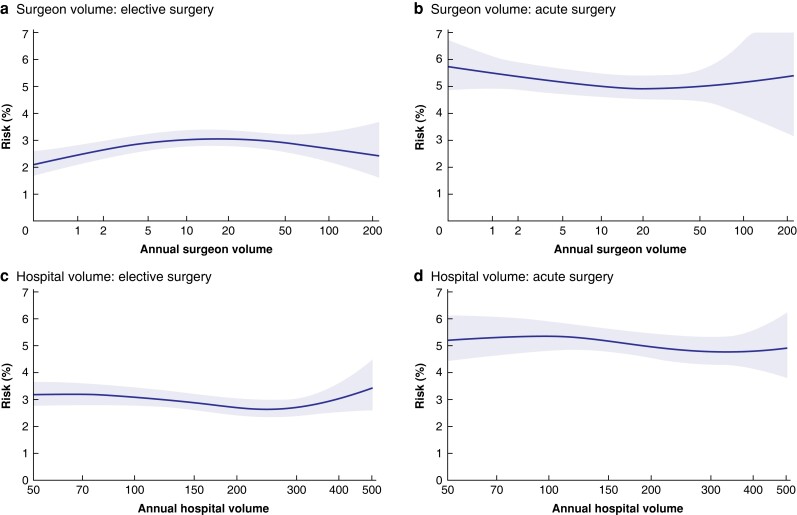
Spline functions for surgical complications in elective and acute procedures, with respect to operative volume for surgeons and hospitals Surgical complication risk for **a** elective and **b** acute operations according to surgeon volume, and **c** elective and **d** acute operations according to hospital volume. Shaded areas represent 95% confidence intervals.

The total rate of surgical complications (bleeding, visceral perforation, bile duct injury, bile leakage, abscesses) was 3.4 per cent in elective and 5.3 per cent in acute surgery. Surgical complication rates were slightly higher for surgeons with annual volumes of between 10 and 33 operations in elective surgery compared with the highest-volume category, although there was no difference between the lowest and highest quartile (*Table 3*). However, no significant difference was evident in acute surgery. There was no difference in surgical complication rate between the volume categories at the hospital level (*Table 3* and *Fig. 3*).

**Table 3:** Generalized estimated equations for surgeon and hospital volumes and surgical outcomes

	Volume per year	Elective surgery*		Acute surgery†	
		OR	*P*	OR	*P*
Surgical complications	Surgeon				
	ȃ≤ 9	1.08 (0.95, 1.23)	0.257	1.08 (0.93, 1.26)	0.312
	ȃ10–19	1.15 (1.01, 1.31)	0.033	1.01 (0.86, 1.18)	0.926
	ȃ20–33	1.16 (1.02, 1.31)	0.025	0.96 (0.82, 1.12)	0.609
	ȃ> 33	1.00 (reference)		1.00 (reference)	
	Hospital				
	ȃ≤136	1.09 (0.95, 1.26)	0.203	1.06 (0.91, 1.24)	0.457
	ȃ137–210	1.07 (0.93, 1.23)	0.334	1.04 (0.91, 1.19)	0.526
	ȃ211–305	0.94 (0.83, 1.06)	0.319	0.95 (0.83, 1.09)	0.474
	ȃ> 305	1.00 (reference)		1.00 (reference)	
Bile duct injury	Surgeon				
	≤ 9	1.41 (1.03, 1.95)	0.033	1.03 (0.71, 1.49)	0.875
	ȃ10–19	1.58 (1.15, 2.17)	0.005	0.88 (0.60, 1.30)	0.528
	ȃ20–33	1.33 (0.98, 1.80)	0.063	0.98 (0.65, 1.48)	0.913
	ȃ> 33	1.00 (reference)		1.00 (reference)	
	Hospital				
	≤ 136	1.75 (1.23, 2.49)	0.002	1.52 (0.95, 2.44)	0.082
	ȃ137–210	1.97 (1.39, 2.81)	<0.001	1.96 (1.25, 2.94)	0.003
	ȃ211–305	1.42 (0.96, 2.09)	0.078	1.84 (1.21, 2.79)	0.004
	ȃ> 305	1.00 (reference)		1.00 (reference)	
Conversion to open surgery	Surgeon				
	ȃ≤ 9	1.35 (1.04, 1.75)	0.023	2.41 (1.91, 3.04)	<0.001
	ȃ10–19	1.39 (1.07, 1.80)	0.014	1.68 (1.33, 2.11)	<0.001
	ȃ20–33	1.26 (1.04, 1.59)	0.020	1.28 (1.05, 1.56)	0.017
	ȃ> 33	1.00 (reference)		1.00 (reference)	
	Hospitalȃ				
	ȃ≤136	2.66 (1.95, 3.63)	<0.001	3.59 (2.96, 4.34)	<0.001
	ȃ137–210	2.10 (1.64, 2.69)	<0.001	3.11 (2.55, 3.79)	<0.001
	ȃ211–305	1.41 (1.14, 1.75)	0.002	1.78 (1.48, 2.14)	<0.001
	ȃ> 305	1.00 (reference)		1.00 (reference)	
30-day mortality	Surgeon				
	ȃ≤ 9	1.74 (0.80, 3.81)	0.164	1.49 (0.83, 2.68)	0.180
	ȃ10–19	1.32 (0.58, 3.02)	0.513	1.44 (0.78, 2.68)	0.248
	ȃ20–33	0.59 (0.22, 1.56)	0.286	1.20 (0.67, 2.18)	0.540
	ȃ> 33	1.00 (reference)		1.00 (reference)	
	Hospital				
	ȃ≤136	2.07 (0.87, 4.95)	0.102	2.53 (1.29, 4.94)	0.007
	ȃ137–210	1.84 (0.74, 4.56)	0.190	2.09 (1.09, 3.98)	0.026
	ȃ211–305	0.77 (0.26, 2.32)	0.640	1.76 (0.88, 3.51)	0.110
	ȃ>305	1.00 (reference)		1.00 (reference)	

Values in parentheses are 95% confidence intervals. The analyses were adjusted for age, sex, and ASA grade. Some *216 and †648 patients were excluded owing to missing values.

The total 30-day complication rate, including surgical complications and all other registered intraoperative and postoperative complications (such as wound infections, thrombosis, cardiac and pulmonary complications, mortality) was 8.5 per cent for elective procedures and 13.3 per cent for acute procedures. The total complication rate was slightly higher for surgeons with annual volumes of 9 or fewer procedures (OR 1.11; *P* = 0.027) and volumes between 10 and 19 (OR 1.12; *P* = 0.017) in elective surgery, but no significant disparity could be seen in the total complication rate between the other groups at either the individual or hospital level (data not shown).

### Bile duct injury

**Fig. 4:**
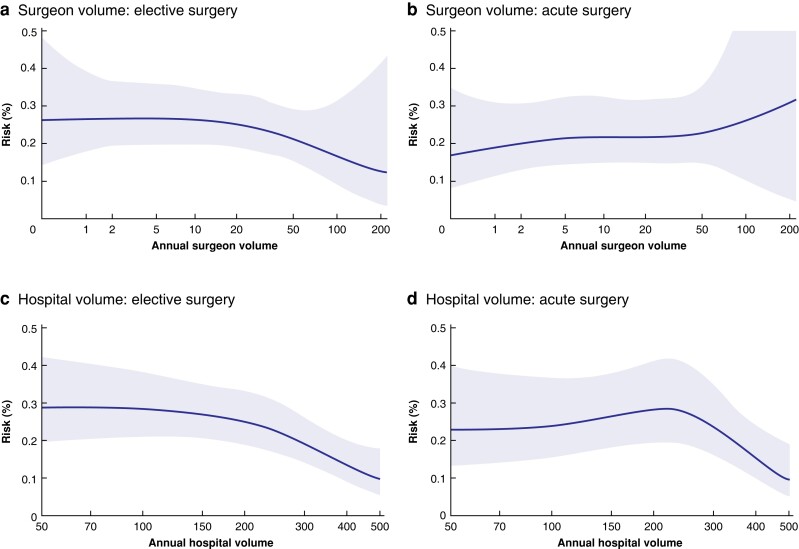
Spline functions for bile duct injury in elective and acute procedures, with respect to operative volume for surgeons and hospitals Bile duct injury risk for **a** elective and **b** acute operations according to surgeon volume, and **c** elective and **d** acute operations according to hospital volume. Shaded areas represent 95% confidence intervals.

The risk of bile duct injury was 0.3 per cent in elective and 0.4 per cent in acute surgery, including any lesion to the bile ducts diagnosed during or after the operation, except for the cystic duct. Surgeons with a low annual volume caused more bile duct injuries in elective surgery. No significant disparity could be seen for acute surgery. Hospitals with low annual volumes had a higher frequency of bile duct injuries, in both elective and acute procedures (*Table 3* and *Fig. 4*). Bile leakage was diagnosed after the operation in 1118 (1.1 per cent) of the elective and 876 (1.6 per cent) of the acute procedures. The leakage was described as leakage from the cystic duct in 441 (0.4 per cent) elective and 361 (0.7 per cent) acute procedures, and from an unknown location in 304 (0.3 per cent) elective and 263 (0.5 per cent) acute operations. No statistically significant correlation between leakage from the cystic duct and operative volume was evident at either the individual or hospital level.

### Conversion to open surgery

**Fig. 5:**
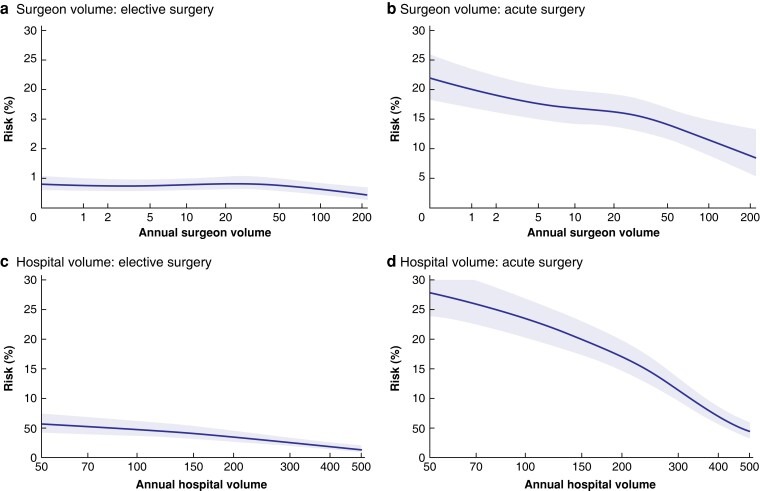
Spline functions for conversion to open surgery in elective and acute procedures, with respect to operative volume for surgeons and hospitals Risk of conversion to open surgery for **a** elective and **b** acute operations according to surgeon volume, and **c** elective and **d** acute operations according to hospital volume. Shaded areas represent 95% confidence intervals.

Surgical approaches in the cohort are recorded in *Table 1*. The risk of an open operation or conversion to open surgery was significantly higher for all quartiles in elective and acute surgery compared with the highest-volume category, for both surgeons and hospitals and, most notably, for acute surgery (*Table 3* and *Fig. 5*).

### Mortality

The overall 30-day mortality rate was 0.05 per cent after elective and 0.33 per cent after acute surgery. It was significantly higher for hospitals with low operative volumes of acute surgery (*Table 3*).

## Discussion

This study has demonstrated that the surgeon’s and hospital’s cholecystectomy volumes have an impact on duration of operation and surgical outcomes. This indicates that gallbladder surgery should be performed by surgeons with high operative volumes at high-volume hospitals, to decrease the frequency of adverse events and improve healthcare for patients with gallstone-related diseases. However, because gallstone surgery forms an important part of surgical education, senior advice and assistance is essential during the learning process, and a balance between high volume and education must be achieved.

According to the present results, high-volume surgeons have shorter operating times and are less prone to convert to open surgery. The same tendency is shown by high-volume hospitals. The association between increased operative volumes for cholecystectomies and decreased operating time, and costs, has been demonstrated previously^18,19^. Conversion to open surgery is associated with significantly longer hospital stay and prolonged recovery time^20^. However, open surgery may be the safest alternative in selected patients for whom laparoscopic access is unsatisfactory, owing to anatomical variations or extensive inflammation. The decision to convert may reflect an ability to reconsider the circumstances and choose a new approach, based on extensive experience with gallstone surgery, but it can also reflect a lack of skills to solve complicated situations laparoscopically.

In this study, low-volume surgeons had more bile duct injuries and slightly more surgical complications than high-volume surgeons in elective surgery, but there was no difference between the volume categories for acute surgery. Low-volume hospitals had a higher frequency of bile duct injuries in both acute and elective operations. The association between high volume and decreased levels of adverse events in acute surgery has been observed in other studies^9,11^. The statistically non-significant results for surgical complications and bile duct injuries in acute surgery are somewhat surprising. They may be explained by residual confounding related to volume and outcome, or the fact that differences in outcomes after acute surgery are influenced to a greater extent by other factors. Previously published studies^21–24^ have suggested that patient characteristics, such as male sex, age, and previous upper abdominal surgery, or the presence of cholecystitis, have a greater impact on outcomes than the surgeon’s operative volume. Although the mortality rate after gallbladder surgery is low, 30-day mortality doubled in hospitals with less extensive volumes. This was also observed in the Scottish population by Harrison *et al*.^12^ in 2012.

Some limitations should be mentioned. The national coverage of 94.5 per cent and the 97 per cent follow-up are strengths of the registry and the study, as well as the magnitude of the database^14,17^. There are, however, obvious limitations with registry-based studies. Intraoperative data should be registered as soon as possible after the operation, but this may vary as a result of different routines. Therefore, incorrect and missing data may be introduced. The complication rate is based on information from the medical records. It is possible that patients with complications are treated at a hospital other than the one in which they underwent surgery. This may be a source of bias when comparing high- and low-volume units, especially for elective operations at private clinics and for acute operations, which are usually performed at the local hospital, even if the patient originates from another part of the country. Hospitals in the same region in Sweden often use the same system for medical records. The estimate of operative volumes in this study is based on the lead operating surgeon. However, the most senior colleague may be registered as the lead surgeon, even if a major part of the operation is performed by a less experienced surgeon. This may also have affected the association between surgeon volume and duration of operation. Because annual volumes are based on the year preceding the cholecystectomy, experienced surgeons may have been classified incorrectly as low-volume surgeons if they mainly assisted colleagues undergoing training. Surgical volumes may also differ over time owing to education, research, parental leave, level of competence of other members of the surgical team, surgical equipment, and reorganization. Furthermore, surgeons may have acquired advanced skills from performing other surgical procedures.

Patient age, sex, and ASA grade were considered as potential confounders and included in the outcome analysis. However, BMI was not adjusted for. The registry’s BMI variable was mandatory only from 2010, so there is a lot of missing information in the cohort. Older age and male sex are known risk factors for a more complex procedure, and are common predictors of conversion to open surgery^25,26^. ASA grade indirectly includes BMI and should also be taken into consideration, as hospitals with elective profiles, such as private clinics, generally operate on healthier patients. The reason for conversion and other patient co-morbidities were not included in the analysis. Furthermore, the severity of the inflammation in acute surgery was not registered, which may be a confounding factor.

The spline diagrams show wide confidence intervals for the lowest- and highest-volume categories. Case mix and individual surgical skill certainly result in variation at the individual level. For hospitals, smaller units might have a few active, high-volume surgeons contributing to the total volume, and other hospitals, such as universities, may have many low-volume surgeons. A high individual volume, as well as experience with more complicated gallstone operations, can be maintained by performing both elective and acute procedures, and by avoiding dividing gallstone surgery between multiple units and individuals. In addition, gallbladder surgery is, and will probably continue to be, an important part of surgical education. The volume–outcome relationship demonstrated in this study highlights the importance of having senior advice available when needed, especially for surgeons with limited experience of gallstone surgery.

Based on the present results, it is difficult to recommend a minimum annual volume of cholecystectomies. For most variables, there seems to be a decrease in adverse events, or at least a flattened curve, for the two highest-volume categories. The definition of low volume as fewer than 20 operations per individual and fewer than 211 operations per hospital each year is comparable to that in other studies^8,10–13,21,22^ and might be an amount to strive for. Here, less than 50 per cent of all surgeons and hospitals achieved this. It would not be realistic to encourage a reduction in the number of surgeons performing cholecystectomies in Sweden based on these limits. However, the results emphasize the importance of procedure-dedicated and accredited surgeons, as well as the development of hospitals with considerable experience of treating and optimizing patients with gallstone-related diseases. Previous studies of more advanced surgical procedures have shown that high-volume institutions have better outcomes, not only because of higher volumes, but also owing to the presence of multidisciplinary teams with well established routines, standardized perioperative care, and specialists available around the clock^6^. Important factors to strive for in gallstone surgery are efficient day care for elective surgery, routines for acute cholecystectomies (to prioritize and operate on patients without delay), and competence in the perioperative removal of common bile duct stones. However, centralizing healthcare must be carried out taking population density into consideration. Centralization of malignant surgery in Sweden has affected organization such that many university clinics perform limited volumes of benign general surgery, such as cholecystectomies^14^. For gallstone surgery, the referral centre may be a regional or county hospital^27^. This is a way to provide access to a high-volume centre, within a reasonable distance, for the entire population.

These results are based on the Swedish population, geography, and surgical setting, which affects their generalizability. They should be interpreted with caution in countries with other healthcare structures. Nevertheless, the study emphasizes the importance of surgical volume at the individual and hospital level in less complex procedures, such as cholecystectomies.

## Data Availability

The research data is available from the corresponding author upon reasonable request.
